# The Development of an Interactive Voice Response Survey for Noncommunicable Disease Risk Factor Estimation: Technical Assessment and Cognitive Testing

**DOI:** 10.2196/jmir.7340

**Published:** 2017-05-05

**Authors:** Dustin G Gibson, Brooke A Farrenkopf, Amanda Pereira, Alain B Labrique, George William Pariyo

**Affiliations:** ^1^ Johns Hopkins Bloomberg School of Public Health Department of International Health Baltimore, MD United States

**Keywords:** interactive voice response, noncommunicable disease, survey methodology, public health surveillance, cellular phone, risk factors

## Abstract

**Background:**

The rise in mobile phone ownership in low- and middle-income countries (LMICs) presents an opportunity to transform existing data collection and surveillance methods. Administering surveys via interactive voice response (IVR) technology—a mobile phone survey (MPS) method—has potential to expand the current surveillance coverage and data collection, but formative work to contextualize the survey for LMIC deployment is needed.

**Objective:**

The primary objectives of this study were to (1) cognitively test and identify challenging questions in a noncommunicable disease (NCD) risk factor questionnaire administered via an IVR platform and (2) assess the usability of the IVR platform.

**Methods:**

We conducted two rounds of pilot testing the IVR survey in Baltimore, MD. Participants were included in the study if they identified as being from an LMIC. The first round included individual interviews to cognitively test the participant’s understanding of the questions. In the second round, participants unique from those in round 1 were placed in focus groups and were asked to comment on the usability of the IVR platform.

**Results:**

A total of 12 participants from LMICs were cognitively tested in round 1 to assess their understanding and comprehension of questions in an IVR-administered survey. Overall, the participants found that the majority of the questions were easy to understand and did not have difficulty recording most answers. The most frequent recommendation was to use country-specific examples and units of measurement. In round 2, a separate set of 12 participants assessed the usability of the IVR platform. Overall, participants felt that the length of the survey was appropriate (average: 18 min and 31 s), but the majority reported fatigue in answering questions that had a similar question structure. Almost all participants commented that they thought an IVR survey would lead to more honest, accurate responses than face-to-face questionnaires, especially for sensitive topics.

**Conclusions:**

Overall, the participants indicated a clear comprehension of the IVR-administered questionnaire and that the IVR platform was user-friendly. Formative research and cognitive testing of the questionnaire is needed for further adaptation before deploying in an LMIC.

## Introduction

The increasing rise in mobile phone ownership and access in low- and middle-income countries (LMICs)—from 1 billion in 2000 to 6 billion in 2012—introduces the opportunity to transform the current paradigm of surveillance activities and to potentially improve the efficiency and timeliness of data collection and reporting [1]. One such opportunity, mobile phone surveys (MPS), offers several potential advantages over traditional household-based surveys. These advantages include real-time data entry to enable timely data analysis, survey delivery that is less demanding on financial and human resources, and anonymity of responses [2]. With the growing noncommunicable disease (NCD) burden [3], there is a subsequent greater need to collect and utilize data to guide public health programs and curb the global NCD epidemic [4].

Interactive voice response (IVR) technology is one of the several options for conducting an MPS. IVR utilizes a prerecorded questionnaire that is administered over the phone [5]. Participants select responses via touchtone keypad or voice recognition software. Responses are immediately submitted to either Web-based databases or internal servers to enable timely data synthesis and analysis [6]. IVR counters a key challenge of short message service (SMS) surveys: the requirement of literate populations.

In adapting a household-administered questionnaire to an IVR survey, cognitive testing of the IVR questionnaire and assessing its usability becomes imperative given IVR’s limitations; especially due to its self-administered nature where respondents are not afforded an opportunity to ask any clarifying questions. In survey development, cognitive testing is frequently applied in order to identify questions that respondents have difficulty comprehending and to assure that the questions adequately capture information as intended [7,8]. Results from cognitive testing can guide question wording and formatting, leading to greater understandability and accuracy in survey responses [9].

The two objectives of this pilot study were to (1) cognitively test and identify challenging questions in an NCD risk factor questionnaire administered via IVR and (2) assess the usability of the IVR platform and identify future challenges for its implementation in LMICs. The findings from this research will be used to revise the questionnaire and IVR platform before conducting a similar series of formative activities in each LMIC where the IVR survey will be deployed.

## Methods

### Questionnaire Development

As part of the Bloomberg Philanthropies Data for Health Initiative (BD4HI) [10], experts in survey methodology, NCDs, and mobile health convened in June 2015 to develop an NCD risk factor questionnaire that could be adapted to an MPS and used to collect population-representative estimates from LMICs [11]. Questions were selected from standardized household surveys such as WHO STEPwise Surveillance, Tobacco Questions for Surveys, and the Behavioral Risk Factor Surveillance System [12-14]. Questions that mapped to indicators in the Global Monitoring Framework for NCDs and that covered the 4 main risk factors for NCDs (physical activity, alcohol consumption, tobacco use, and diet) were preferred [15]. Questions were selected for an IVR survey independent of their perceived suitability to the IVR modality. This produced a *beta* version of the questionnaire that was adapted to VOTO mobile’s IVR platform—a Ghana-based organization that works to develop MPS systems [16]—and used to cognitive test the IVR survey and to assess its usability (see Multimedia Appendices 1 and 2).

**Figure 1:**
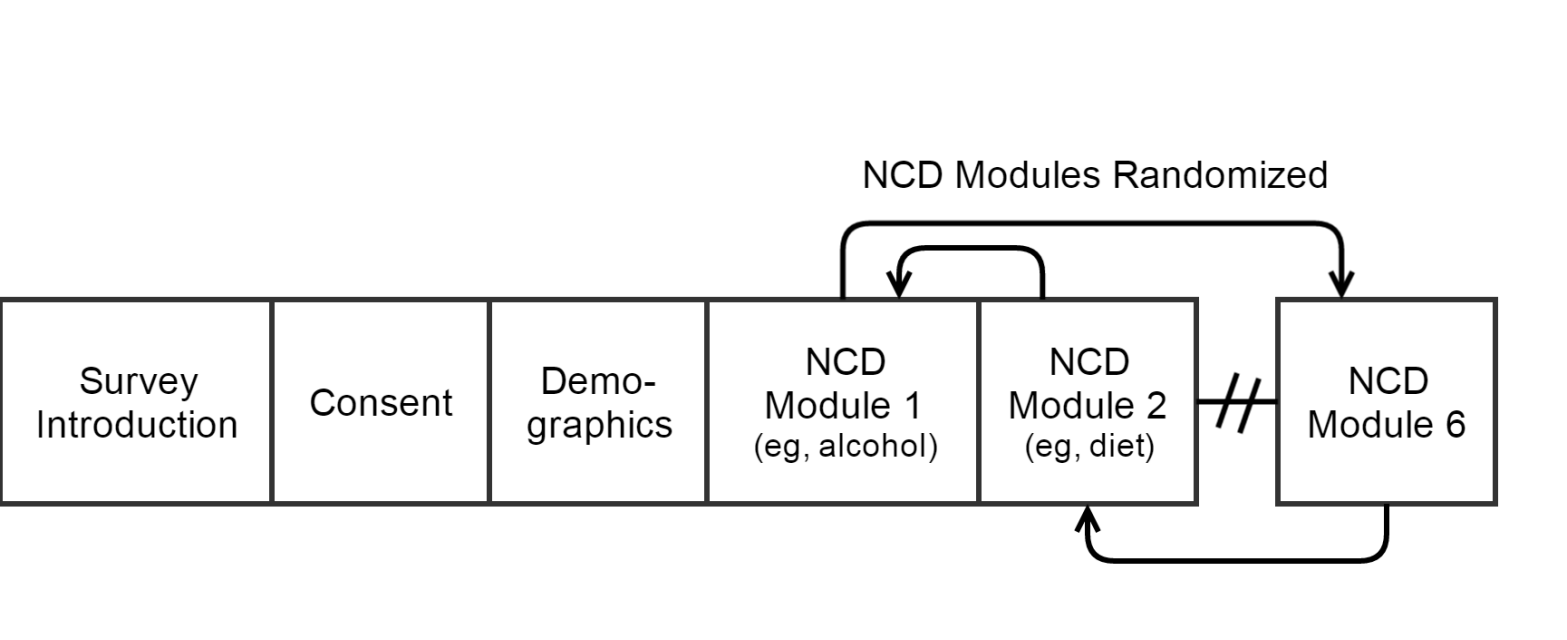
Interactive voice response (IVR) survey design. NCD: noncommunicable disease.

The IVR survey included a brief introduction, and was followed by a question asking for assent to participate, demographic questions, and NCD modules (Figure 1). Modules were a series of topically similar questions such as alcohol consumption, tobacco use, and dietary intake (Table 1). The IVR platform was programmed to randomize the delivery order of the NCD modules. For each question, the IVR survey was programmed so that respondents could repeat the question by pressing the asterisk, “star key,” on the mobile phone.

**Table 1:** Number and source of questions included in each module by round of pilot testing.

Module	Round 1 n questions	Round 2 n questions	Source survey
Introduction or consent	1	1	-
Demographics	5	6	-
Tobacco	2	2	TQS
Alcohol	3	3	STEPS
Diet	10	10	STEPS
Diabetes and blood pressure medication	4	4	STEPS
Physical activity	12	-	IPAQ
Physical activity	-	20	GPAQ
Lifestyle	6	-	STEPS
Total N questions	43	46	

### Participants

Participants were eligible for inclusion in the pilot study if they were proficient in English and identified as being a native of any country within Africa, Asia, Latin America, or South America. Participants were excluded if they were aged under 21 years or had a hearing impairment. The study received ethical approval from the JHSPH Institutional Review Board (IRB). Study participants provided oral consent and were compensated for their participation with a US $10 gift-card to a local coffee shop.

### Data Collection and Analysis

We conducted two rounds of pilot testing, with different participants in each round, to gain clearer understanding of the participants’ thought processes (round 1) and to assess the usability of the IVR survey (round 2). There were minor differences in the questions and modules between the two rounds (Table 1). Participants listened to the IVR survey on a Samsung Convoy 3 mobile phone provided by the study team. This simple phone was selected to serve as a proxy for the types of phones commonly used in LMICs. Participants in each round of pilot testing were instructed to answer the IVR survey honestly and to think about how this survey could be improved if administered in their country of origin.

#### Round 1: Cognitive Testing

In order to minimize recall bias, participants were administered the modules one at a time. After each module, the IVR survey was paused and participants were cognitively tested through active probing to assess their understanding of each question and to identify specific areas of misunderstanding. Representative examples of questions asked of participants in Round 1 are found in Textbox 1.

NCD questions and modules were scored for their comprehensibility, which was considered “high” if >75% of the participants did not express concern over the introduction or question content overall throughout the module; “medium” if 51-75% of participants expressed no concern; and “low” if <50% of participants found no difficulty.

#### Round 2: Assessment of IVR Platform Usability

During round 2, groups of 2-3 participants listened to the IVR survey in its entirety and were then asked about the IVR usability and any overall concerns with the survey (see Textbox 2). Participants were encouraged to vocalize any comments about the survey’s wording, length, understandability, and ways to improve survey performance if administered nationally in an LMIC setting**.** Comments on the survey length, the survey’s introduction, and features they liked or did not like about the IVR platform were compiled. Following the focus groups, participants were individually administered the GPAQ physical activity questions through IVR and were cognitively tested to assess their understanding using methods similar to those employed in round 1. No other NCD modules were cognitively tested during round 2.

**Examples of questions asked during pilot testing in round 1.**

Round 1: Cognitive testing

- Was this question clear?
- Were there any words or phrases that were difficult to understand?
- What does the word, “XXX” mean to you?
- How confident were you in your answer?
- Do you foresee any challenges asking this question in an LMIC?

**Examples of questions asked during pilot testing in round 2.**

Round 2: Usability of interactive voice response (IVR)

- What factors would cause you to be more or less likely to participate in a similar mobile phone administered health or NCD surveys?
- Comment on your reaction and experiences during the IVR survey or how you would react if you got such a survey in the future?
- How do you expect people in your country or community would react if they received such a survey on their mobile phones?
- The modules were randomized—do you think there are any issues to consider in randomizing questions in your home country?

## Results

From January to February 2016 in Baltimore, MD, a total of 24 participants were pilot tested, with 12 participants in each of the 2 rounds.

### Cognitive Testing: Round 1

In Round 1 (n=12), the median participant’s age and years of education completed were 27 years old (IQR: 25-30 years; range: 22-56 years) and 15 years (IQR: 13-18 years; range: 9-20 years), respectively (Table 2). Cognitive testing through individual interviews with the participants identified that the examples that were used in the question to help respondents in answering (eg, “I would now like to ask you about smoking tobacco, including cigarettes, cigars, and pipes.”) as a common area of concern across the NCD modules. All 12 participants (100%) commented that the provided examples for diet, physical activity, tobacco, and alcohol should be specific to the country where the survey is being conducted.

**Table 2:** Demographic characteristics of participants by round.

Demographic characteristics		Round 1 n=12	Round 2 n=12		Total n=24	
**Sex, n (%)**						
	Male	4 (33)		4 (33)		8 (33)
	Female	8 (67)		8 (67)		16 (67)
**Region of birth, n (%)**						
	Africa	2 (17)		8 (67)		10 (42)
	South Asia	5 (42)		1 (8)		6 (25)
	Central and East Asia	2 (17)		1 (8)		3 (13)
	Latin America	2 (17)		1 (8)		3 (13)
	South America	1 (8)		1 (8)		2 (8)
Age in years, median (IQR)		27 (25-30)	28.5 (26-33)		27 (25-31)	
Education in years, median (IQR)		15 (13-18)	18 (14-19)		16 (13-18)	

In addition to this survey-wide comment, several questions were problematic for participants. These questions and their respective challenges are listed below and summarized in Table 3.

#### Urban or Rural Setting

(In your home country) do you live in a rural or urban area? If you live in a rural area, press 1. If you live in an urban area, press 3.

This proved to be one of the more challenging questions, with 11 (91.7%) participants raising concern that other people taking this survey may have difficulty distinguishing between and defining urban and rural. Two participants (16.7%) suggested including “peri-urban” as an available response.

#### Education

Not including preschool, how many years of school and full time study have you completed? Please enter the number of years.

All participants had issues with this question, with many reporting that they felt rushed to calculate a response. Four participants (33.3%) recommended removing the word “preschool” and three participants (25%) recommended converting the responses to be categorical (eg, primary school, secondary school, and so on) such that it matches the country’s education system.

#### Alcohol

One drink is equivalent to a 12 ounce beer, a five ounce glass of wine, or a drink with one shot of liquor...

Three participants (25%) did not understand at least one of the terms of measurement used and stressed that in-country research would be necessary to provide the accurate measurement and country-specific examples.

#### Salt Intake

...I would like you to think about all the sources of salt, including ordinary table salt, unrefined salt such as sea salt, iodized salt, salty stock cubes and powders, and salty sauces such as soya sauce or fish sauce.

Approximately 92% (11/12) of participants found this confusing, stating that they were unfamiliar with some of the examples used (eg, participants said soya sauce was not used in their country). One-third of participants (n=4) said there were too many examples listed and recommended against using similar sounding examples or sentence structure.

#### Vegetable Consumption

A serving of vegetables is about a cup of green leafy vegetables or salad or half a cup of cooked or chopped vegetables. How many of these servings of vegetables do you eat on one of those days?

Three participants (25%) expressed confusion with this question. Participants commented that the use of “leafy greens” directed them to include only green vegetables and exclude other vegetables, like carrots.

#### Physical Activity

How much time do you spend doing vigorous-intensity activities at work on a typical day? I will ask you to enter hours followed by minutes. Please enter between 16 & 0 hours now?

After participants reported hours, the question was followed by

Now enter between 59 & 0 minutes.

Half of the participants (n=6) thought that the way time was measured was confusing; by first asking about hours and then minutes. Participants suggested that the question be simplified by only asking about hours.

**Table 3:** Summary of respondents’ assessment of comprehensibility of interactive voice response (IVR) questionnaire.

Module	Level of comprehensibility^a^ (high, medium, low)	Remarks
Demographics	Medium	Participants had low comprehensibility with the questions regarding education and rural or urban settings, but high comprehensibility with questions on age and sex.
Tobacco	High	No challenges other than providing country-specific examples of tobacco.
Alcohol	Medium	Participants had difficulty with units given to measure their alcohol consumption.
Diet	Medium	Participants had low comprehensibility with the salt questions, but high and medium comprehension with the fruit and vegetable questions, respectively.
Blood pressure and diabetes	High	No challenges identified.
Physical activity (IPAQ)	Medium	Participants had difficulty differentiating between levels of activity (moderate vs vigorous). Question structure was repetitive leading to reporting fatigue.
Physical activity (GPAQ)	Low	Participants had difficulty differentiating between levels of activity and with the question structure to estimate their time spent doing physical activity. Question structure was repetitive leading to reporting fatigue.
Lifestyle	High	No challenges identified.

^a^Comprehensibility was considered “high” if >75% of the participants in round 1 of testing did not express concern over the introduction or question content overall throughout the module; “medium” if 51-75% of participants expressed no concern; and “low” if <50% of participants found no difficulty.

### Usability of the IVR Platform: Round 2

Round 2 participants were similar in demographics to those of Round 1; the median participant’s age and years of education completed were 28.5 years old (IQR: 26-33 years; range: 22-35 years) and 18 years (IQR: 14-19 years; range: 4-20 years), respectively (Table 2). Due to skip patterns programmed into the 46-question survey, the average number of questions answered was 36. Participants spent an average of 18 min and 31 s answering the survey (data not shown).

#### Survey Length

Overall, the majority of participants thought that the length of the survey was appropriate, with many participants commenting on the thoroughness of the survey. When asked how long they would be willing to spend completing a survey via mobile phone, participants estimated a range of 10-20 min. Participants were more critical of the length of specific questions and modules than the overall survey. During certain modules, response fatigue was related to confusion over unfamiliar examples, repetitive question structure, and difficulty in understanding the narrator.

#### Survey Introduction

The survey began with an introduction that contained keypress instructions, the expected survey duration, and that the survey was “sponsored by the Ministry of Health.” The majority of participants thought that the introduction and instructions were sufficient to complete the IVR survey. Some participants commented that indicating that the survey was sponsored by the government may affect response rate either positively or negatively depending on the country context. For instance, participants thought that respondents might be more hesitant to partake in a government-sponsored survey during an election period.

#### Survey Features

Participants appreciated the consistent use of key press options for questions that had two answers, such as the Yes or No questions (eg, Press1 for Yes, Press 3 for No). Similarly, participants appreciated being allowed to repeat the question by pressing the asterisk (star) key.

Several participants also commented that the time between providing a response and the narration of the subsequent question was too short. Some participants did not hear the first few words of the next question as they were bringing the phone back to their ear after entering their response.

Nearly all participants experienced confusion with understanding the accent of the narrator selected to record the survey (Ghanaian). Many participants commented that the narrator was monotone and spoke too quickly. Participants recommended that the narrator use inflection to highlight key points of the question.

#### Question Structure

Participants found lengthy questions and modules with similar sentence structure to be repetitive, causing them to lose focus. This was a key issue in the physical activity module, where three similar sets of questions asked about three different levels of physical activity: (1) vigorous physical activity, (2) moderate physical activity, and (3) walking. After answering the vigorous physical activity questions, some participants did not realize that they were being asked new questions about moderate physical activity. Nearly all participants had issues distinguishing between the levels of activity (eg, vigorous vs moderate), and some admitted to realizing that they unintentionally double-counted their activity.

#### Perceived Barriers and Solutions to IVR

When asked about potential barriers to deploying IVR surveys in their birth country, participants mentioned that respondents might be unable to move to a quiet location at the time of the call and may refuse to answer an incoming call with an unfamiliar phone number. Participants also thought that future IVR surveys would benefit from including an incentive as some people might not complete the survey if they were randomly dialed. However, they also stated that requiring a user to input personal information (such as bank account information) via phone would be problematic even with an incentive and may lead to not participating in the survey at all. When told about an airtime incentive that would not request any additional or personal information, all participants agreed that this was a preferable option.

Nearly all participants preferred a survey conducted over the phone rather than in person. Participants appreciated the anonymity of the IVR survey and liked that it felt less personal than a face-to-face interview, establishing a foundation to respond honestly. Participants expressed concern over topics that may be perceived as sensitive or controversial in their home country, including alcohol intake and medical diagnoses. Some participants expressed that respondents might be encouraged to select the answer that supports the more socially accepted, “healthy” option.

## Discussion

### Principal Findings

The IVR platform presents an opportunity to transform current modes of data collection for large-scale household surveys and to potentially improve the quality and utilization of the data [17]. Findings from the two rounds of pilot testing provide guidance to tailor the IVR platform for increased usability among future respondents and to identify challenging questions that need further refinement to ensure that the intended data are being collected correctly.

The common request to have greater in-country influence in question development and structure suggests the need for similar in-country cognitive testing. Conducting key informant interviews and focus group discussions would enable country-specific survey adaptation before national administration. Formative research should focus on selection of local terms to use as examples (eg, types of physical activity, terms of measurement). For some surveys, using different sets of modules with subnational, region-specific terms could enhance understandability and accuracy of responses. Local country-specific adaptation with relevant country examples will be important during future developments of IVR surveys, even if the generic survey is also meant for cross-country comparisons of key indicators. For instance, in low literacy settings, the concept of time down to minutes may be difficult to elicit and one may use half an hour or quarter of an hour blocks of time to describe duration of activities being investigated [18].

Formative research should further focus on selecting narrators that are native to countries and regions, where dialects may vary throughout the country. Guidance to have the narrator speak slowly and intonate specific words, strategically selected by the questionnaire development team to emphasize key details, should be integral during the audio recording phase [19]. Greater intonation and question structure variety can also make an IVR survey more personal, while still attaining the benefits of anonymity [20]. Research to determine topics perceived as sensitive or personal would aid in determining questions where participants place greater importance on confidentiality. A reminder of confidentiality before modules with sensitive or personal content could aid in participant retention and truthfulness (eg, “As a reminder, this survey is confidential”) [21]. Consideration should also be given to the age and gender of the narrator. In Ghana, researchers found that IVR surveys narrated by a female led to a higher response rate [22].

One of the most promising findings from the testing was the overall acceptance of the survey length (average: 18 min and 31 s) and the appreciation of instruction thoroughness. This suggests that surveys of similar length may be a feasible option to complement existing surveillance methods, but they require further empirical testing. Participants’ appreciation of the thoroughness of the question prompts and module introductions, even at the expense of increasing the length of the survey, show that it may not always be necessary to compromise detail to minimize overall survey length as long as overall time duration is kept reasonable. Participants did not like the feeling of being rushed to answer the question, which supports lengthening the time provided to select a response. In future formative work, it will be important to test out various durations of pauses in-between questions so that participants will not *miss out* on hearing part of the question.

When discussing potential barriers to IVR surveys, considerations on the participant’s readiness to complete the survey should be made. Some participants were concerned that future respondents would not be available to complete the survey upon receiving the call. This presents the opportunity to inform the respondents in advance, via SMS message for example, that they will receive a call from an unknown phone number to administer the survey. Introducing a technology to select a window of time that is best to receive the call could also improve response rate.

With some participants raising concern over the public opinion of government sources and willingness to partake in its surveys, formative research should evaluate the perception and possibilities of text to include in the survey introduction section early on at connection. The introduction should convey the message that this is an officially approved or sanctioned survey being implemented by a nationally recognized public health agency or research institution. The mention of such a neutral agency or public institution by name and that the survey would serve a public health common good through information for better planning could improve participant willingness to continue with the survey. Response rate could also be improved by involving a well-known, popular, and respected figure, such as an athlete, to narrate the survey. To further improve response rate, future research should examine the effectiveness of overall and demographic-specific incentives to improve generalizability of the survey’s study population.

### Limitations

This study has several limitations. First, our sample was primarily composed of students and fellows at a single university, who have knowledge on public health topics and surveillance methods. Therefore, the participants in this study may have had less difficulty in understanding the questionnaire as compared with other members in an LMIC community. Similarly, many participants were not asked follow-up questions about risk factors or health conditions, therefore possibly resulting in a shorter survey than what might be observed if sent to a sample representative of the community. Second, our study sample was relatively young in age and therefore may be more adept at using a mobile phone. The questionnaire will need to be cognitively tested within each country and among a wide range of demographic groups before its deployment. Third, we did not collect information on how long the participant lived in their native country. This has potential implications on our findings as we asked participants to frame several of their responses through the lens of an LMIC survey participant.

### Conclusions

Overall, participants did not have difficulty with understanding the questions and recording their responses. Most participants appreciated the anonymity of the IVR survey, stating that in comparison with face-to-face interviews, it encourages honest and accurate responses. Participants also felt that the length of the survey was appropriate and expressed a preference to have instructions explained thoroughly.

Incorporating the recommendations common among participants and conducting formative research will help develop an NCD survey that can be administered via IVR, particularly around the selection of country-specific examples and narrators to improve understandability. This shows that IVR may be an appropriate vehicle to administer timely, resource-efficient risk factor surveillance among populations in LMIC settings.

## Appendix

Multimedia Appendix 1 Questionnaire used for cognitive testing (round 1).

Multimedia Appendix 2 Questionnaire used for usability assessment (round 2).

## Abbreviations

BRFSS: Behavioral Risk Factor Surveillance System
CDC: Centers for Disease Control and Prevention
GPAQ: Global Physical Activity Questionnaire
IPAQ: International Physical Activity Questionnaire
IQR: Interquartile range
IVR: interactive voice response
JHSPH: Johns Hopkins Bloomberg School of Public Health
LMIC: low- and middle-income country
MPS: mobile phone survey
NCD: noncommunicable disease
SMS: short message service
WHO: World Health Organization
TQS: Tobacco Questions for Surveys
STEPS: The WHO STEPwise approach to Surveillance
