# Supplementary material for: The Development of an Interactive Voice Response Survey for Noncommunicable Disease Risk Factor Estimation: Technical Assessment and Cognitive Testing
Source: J Med Internet Res. 2017 May 5;19(5):e112. doi: 10.2196/jmir.7340 (PMC5438455; doi:10.2196/jmir.7340)
Supplement: Multimedia Appendix 1 [file jmir_v19i5e112_app1.pdf]

Appendix 1. Questionnaire used for cognitive testing (Round 1)

#	Module	Question
	Introduction	*Hello. This is an automated questionnaire you were informed about earlier from the Ministry of Health. This interview will take no more than 20 minutes of your time. Any information you share will be kept confidential and private, and will only be used to understand more about the health of our country's population. To answer each question, I will ask you to press a number - for example, 1 for YES, and 3 for NO, or to answer a question with a number - like 12 or 22. You can hear a question again at any time by pressing the STAR button - located to the left of the ZERO at the bottom of your phone.*
1	Introduction	Please move to a quiet location, if you can, and press 1 to continue the interview.
2	Demographics	Are you MALE or FEMALE? If you are male, press 1. If you are female, press 3.
3	Demographics	How old are you? Please enter your age. If you don't know exactly, please make your best guess. If you still can't provide an answer, press ZERO.
4	Demographics	In your home country, did you live in a rural or urban area? If you live in a rural area, press 1. If you live in an urban area, press 3.
5	Demographics	Not including pre-school, how many years of school and full time study have you completed? Please enter the number of years
	Tobacco	*I would now like to ask you about smoking tobacco, including cigarettes, cigars and pipes. Please do not include smokeless tobacco or e-cigarettes at this time. As a reminder, you can press the * key for any question that you need to have repeated and you can press ZERO to skip any question you prefer not to answer.*
6	Tobacco	Do you currently smoke tobacco? If you smoke tobacco DAILY, press 1. If you smoke tobacco LESS THAN DAILY, press 2. If you don't smoke tobacco AT ALL, press 3.
	Tobacco	*The next question is about using smokeless tobacco, such as snuff, chewing tobacco, and dip. Smokeless tobacco is tobacco that is not smoked, but is sniffed through the nose, held in the mouth, or chewed.*
7	Tobacco	Do you currently use smokeless tobacco? If you use smokeless tobacco DAILY, press 1. If you use smokeless tobacco LESS THAN DAILY, press 2. If you do not use smokeless tobacco AT ALL, press 3.
	Alcohol	*Now, I would like to ask you some questions about alcohol use. As a reminder, you can press the * key for any question that you need to have repeated and you can press ZERO to skip any question you prefer not to answer.*
8	Alcohol	Have you EVER drank any alcohol such as beer, wine, or spirits? If YES, press 1. If NO, press 3.
9	Alcohol	During the last 30 days, did you drink any alcohol? If YES, press 1. If NO, press 3.
10	Alcohol	One drink is equivalent to a 12 ounce beer, a twelve ounce glass of wine, or a drink with one shot of liquor. Considering all types of alcoholic beverages, how

		many times during the last 30 days did you have 6 or more drinks in a single drinking occasion? Please enter the number of days from 1 to 30.
	Diet	*With the next questions, I would like to learn more about the foods that you eat. As a reminder, you can press the * key for any question that you need to have repeated and you can press ZERO to skip any question you prefer not to answer.*
11	Diet	In the past week, have you eaten any fruit? If YES, press 1. If NO, press 3.
12	Diet	In a typical week, on how many days do you eat fruit? Please enter the number of days from 1 up to a maximum of 7.
13	Diet	A serving of fruit is a medium sized apple, banana or orange or half a cup of cooked or chopped fruit. How many servings of fruit do you eat on one of those days? Please enter the number of servings from 1 up to a maximum of 50.
14	Diet	In the past week, have you eaten any VEGETABLES? If YES, press 1. If NO, press 3.
15	Diet	In a typical week, on how many days do you eat vegetables? Please enter the number of days from 1 up to a maximum of 7.
16	Diet	A serving of vegetables is about a cup of green leafy vegetables or salad or half a cup of cooked or chopped vegetables. How many of these servings of vegetables do you eat on one of those days? Please enter the number of servings from 1 up to a maximum of 50.
	Diet	*With the next questions, I would like to learn more about the salt you eat. I would like you to think about all the sources of salt, including ordinary table salt, unrefined salt such as sea salt, iodized salt, salty stock cubes and powders, and salty sauces such as soya sauce or fish sauce. As a reminder, you can press the * key for any question that you need to have repeated and you can press ZERO to skip any question you prefer not to answer.*
17	Diet	How often do you add salt or a salty sauce such as soya sauce to your food right before you eat it or as you are eating it? If ALWAYS, press 1. If OFTEN, press 2. If SOMETIMES, press 3. If RARELY, press 4. If NEVER, press 5.
18	Diet	How often is salt, salty seasoning or a salty sauce added in cooking or preparing foods in your household? If ALWAYS, press 1. If OFTEN, press 2. If SOMETIMES, press 3. If RARELY, press 4. If NEVER, press 5. If you DON'T KNOW, press 7.
19	Diet	How often do you eat food with a lot of salt in it such as packaged salty snacks, canned salty food such as pickles, salty food prepared at a fast food restaurant, cheese, bacon and processed meat? If ALWAYS, press 1. If OFTEN, press 2. If SOMETIMES, press 3. If RARELY, press 4. If NEVER, press 5.
20	Diet	Do you currently do anything on a regular basis to control your salt intake? If YES, press 1. If NO, press 3.
	Physical Activity	*I am going to ask you about the time you spent being physically active in a typical week. Think about the activities you do at work, as part of your house work, to get from place to place, and in your spare time for recreation, exercise, or sport.*

		Now, think about all the vigorous activities which take hard physical effort that you do in a typical week.<br><br>*Vigorous physical activities make you breathe much harder than normal and may include heavy lifting, digging, aerobics, or fast bicycling. Think only about those physical activities that you do for at least 10 minutes at a time. As a reminder, you can press the * key for any question that you need repeated and you can press ZERO for any question that you prefer not to answer.*
21	Physical Activity	Did you do any VIGOROUS physical activity on at least one day last week? If YES, press 1. If NO, press 3.
22	Physical Activity	In a typical week, on how many days do you do vigorous physical activities? Please enter the number of days from 1 up to a maximum of 7.
23	Physical Activity	How many hours do you usually spend doing vigorous physical activities on one of those days? Please enter the number of hours from 1 up to a maximum of 24. If you prefer to answer in minutes, press 88. Otherwise, please enter the number of hours
24	Physical Activity	How many minutes do you usually spend doing vigorous physical activities on one of those days? Please enter the number of minutes from 1 up to a maximum of 999.
	Physical Activity	*Now think about activities which take MODERATE physical effort that you do in a typical week. Moderate physical activities make you breathe somewhat harder than normal and may include carrying light loads, or bicycling at a regular pace. Do not include walking. Again, think about only those physical activities that you did for at least 10 minutes at a time.*
25	Physical Activity	Did you do MODERATE physical activity on at least one day last week? If YES, press 1. If NO, press 3.
26	Physical Activity	In a typical week, on how many days do you do moderate physical activities? Please enter the number of days from 1 up to a maximum of 7.
27	Physical Activity	How many hours do you usually spend doing moderate physical activities on one of those days? Please enter the number of hours from 1 up to a maximum 24. If you prefer to answer in minutes, press 88. Otherwise, please enter the number of hours
28	Physical Activity	How many minutes do you usually spend doing moderate physical activities on one of those days? Please enter the number of minutes from 1 up to a maximum of 999.
	Physical Activity	*Now think about the time you spent walking in a typical week. This includes at work and at home, walking to travel from place to place, and any other walking that you might do solely for recreation, sport, exercise, or leisure.*
29	Physical Activity	In the past week, have you walked for at least 10 minutes at a time? If YES, press 1. If NO, press 3.

30	Physical Activity	In a typical week, on how many days do you walk for at least 10 minutes at a time? Please enter the number of days from 1 up to a maximum of 7.
31	Physical Activity	How many hours do you usually spend walking on one of those days? Please enter the number of hours from 1 up to a maximum of 24. If you prefer to answer in minutes, press 88. Otherwise, please enter the number of hours
32	Physical Activity	How many minutes do you usually spend walking on one of those days? Please enter the number of minutes from 1 up to a maximum of 999.
		*I am now going to ask you questions about medical conditions and current medications. As a reminder, you can press the * key for any question that you need repeated and you can press ZERO for any question that you prefer not to answer.*
33	BP and Diabetes	Have you EVER been told by a doctor or other health worker that you have raised or high blood pressure or that you have hypertension? If YES, press 1. If NO, press 3
34	BP and Diabetes	In the last two weeks, have you taken any drugs or medication for raised or high blood pressure that was prescribed to you by a doctor or other health worker? If YES, press 1. If NO, press 3.
35	BP and Diabetes	Have you EVER been told by a doctor or other health worker that you have raised or high blood sugar or that you have diabetes? If YES, press 1. If NO, press 3.
36	BP and Diabetes	In the last two weeks, have you taken any drugs or medications such as insulin for diabetes that was prescribed to you by a doctor or other health worker? If YES, press 1. If NO, press 3.
37	Lifestyle	Now I will ask you to think about the last year. In the last year have you seen a doctor or health worker about your health? Press 1 for YES, and Press 3 for NO.
		I will now ask you five questions about advice a doctor may have given you. During the LAST YEAR, has a doctor or other health worker advised you to do any of the following?
38	Lifestyle	Quit using tobacco or not to start. If YES, press 1. If NO, press 3.
39	Lifestyle	Reduce salt in your diet. If YES, press 1. If NO, press 3.
40	Lifestyle	Eat at least five servings of fruit and or vegetables each day. If YES, press 1. If NO, press 3.
41	Lifestyle	During the LAST YEAR, has a doctor or other health worker advised you to do any of the following? Start or do more physical activity. If YES, press 1. If NO, press 3.
42	Lifestyle	Maintain a healthy body weight or lose weight. If YES, press 1. If NO, press 3.
		The survey is now complete. Thank you very much for your time in answering these questions for the Ministry of Health. It will help us better understand the health of our country.
