# Supplementary material for: The Development of an Interactive Voice Response Survey for Noncommunicable Disease Risk Factor Estimation: Technical Assessment and Cognitive Testing
Source: J Med Internet Res. 2017 May 5;19(5):e112. doi: 10.2196/jmir.7340 (PMC5438455; doi:10.2196/jmir.7340)
Supplement: Multimedia Appendix 2 [file jmir_v19i5e112_app2.pdf]

Appendix 2. Questionnaire used for round 2

#	Module	Question
	Introduction	*Hello. This is an automated questionnaire you were informed about earlier from the Ministry of Health. This interview will take no more than 20 minutes of your time. Any information you share will be kept confidential and private, and will only be used to understand more about the health of our country's population. To answer each question, I will ask you to press a number - for example, 1 for YES, and 3 for NO, or to answer a question with a number - like 12 or 22. You can hear a question again at any time by pressing the STAR button - located to the left of the ZERO at the bottom of your phone.*
1	Introduction	Please move to a quiet location, if you can, and press 1 to continue the interview.
2	Demographics	Are you MALE or FEMALE? If you are male, press 1. If you are female, press 3.
3	Demographics	How old are you? Please enter your age. If you don't know exactly, please make your best guess. If you still can't provide an answer, press Zero.
4	Demographics	In your home country, did you live in a rural or urban area? If you live in a rural area, press 1. If you live in an urban area, press 3.
5	Demographics	Not including pre-school, how many years of school and full time study have you completed? Please enter the number of years
6	Demographics	How many mobile phone numbers do you use on a regular basis?
7	Demographics	How many people aged 18 years or older regularly use this mobile phone number?
	Tobacco	*I would now like to ask you about smoking tobacco, including cigarettes, cigars and pipes. Please do not include smokeless tobacco or e-cigarettes at this time. As a reminder you can press the * key for any question that you need to have repeated and you can press zero to skip any question you prefer not to answer*
8	Tobacco	Do you currently smoke tobacco? If you smoke tobacco DAILY, press 1. If you smoke tobacco LESS THAN DAILY, press 2. If you don't smoke tobacco AT ALL, press 3.
	Tobacco	*The next question is about using smokeless tobacco, such as snuff, chewing tobacco, and dip. Smokeless tobacco is tobacco that is not smoked, but is sniffed through the nose, held in the mouth, or chewed.*
9	Tobacco	Do you currently use smokeless tobacco? If you use smokeless tobacco DAILY, press 1. If you use smokeless tobacco LESS THAN DAILY, press 2. If you do not use smokeless tobacco AT ALL, press 3.
	Alcohol	*Now, I would like to ask you some questions about alcohol use. As a reminder you can press the * key for any question that you need to have repeated and you can press zero to skip any question you prefer not to answer*
10	Alcohol	Have you EVER drank any alcohol such as beer, wine, spirits? If YES, press 1. If NO, press 3. As a reminder, you can press the * key for any question that you need to have repeated.

11	Alcohol	During the last 30 days, did you drink any alcohol? If YES, press 1. If NO, press 3.
12	Alcohol	One drink is equivalent to a 12 ounce beer, a twelve ounce glass of wine, or a drink with one shot of liquor. Considering all types of alcoholic beverages, how many times during the last 30 days did you have 6 or more drinks in a single drinking occasion? Please enter the number of days from 1 to 30
	Diet	*With the next questions, I would like to learn more about the foods that you eat. As a reminder you can press the * key for any question that you need to have repeated and you can press zero to skip any question you prefer not to answer*
13	Diet	In the past week, have you eaten any fruit. If YES, press 1, if NO press 3
14	Diet	In a typical week, on how many days do you eat fruit? Please enter the number of days from 1 up to a maximum of 7.
15	Diet	A serving of fruit is a medium sized apple, banana or orange or half a cup of cooked or chopped fruit. How many servings of fruit do you eat on one of those days? Please enter the number of servings from 1 up to a maximum of 50.
16	Diet	In the past week, have you eaten any vegetables? If YES press 1, if NO press 3.
17	Diet	In a typical week, on how many days do you eat vegetables? Please enter the number of days from 1 up to a maximum of 7
18	Diet	A serving of vegetables is about a cup of green leafy vegetables or salad or half a cup of cooked or chopped vegetables. How many of these servings of vegetables do you eat on one of those days? Please enter the number servings from 1 to a maximum of 50
	Diet	*With the next questions, I would like to learn more about the salt you eat. I would like you to think about all the sources of salt, including ordinary table salt, unrefined salt such as sea salt, iodized salt, salty stock cubes and powders, and salty sauces such as soya sauce or fish sauce. As a reminder you can press the * key for any question that you need to have repeated and you can press 0to skip any question you prefer not to answer*
19	Diet	How often do you add salt or a salty sauce such as soya sauce to your food right before you eat it or as you are eating it? If ALWAYS, press 1. If OFTEN, press 2. If SOMETIMES, press 3. If RARELY, press 4. If NEVER, press 5.
20	Diet	How often is salt, salty seasoning or a salty sauce added in cooking or preparing foods in your household? If ALWAYS, press 1. If OFTEN, press 2. If SOMETIMES, press 3. If RARELY, press 4. If NEVER, press 5. If you DON'T KNOW, press 7.
21	Diet	How often do you eat food with a lot of salt in it such as packaged salty snacks, canned salty food such as pickles, salty food prepared at a fast food restaurant, cheese, bacon and processed meat? If ALWAYS, press 1. If OFTEN, press 2. If SOMETIMES, press 3. If RARELY, press 4. If NEVER, press 5.
22	Diet	Do you currently do anything on a regular basis to control your salt intake? If YES, press 1. If NO, press 3.

	BP and Diabetes	*I am now going to ask you questions about medical conditions and current medications. As a reminder you can press the * key for any question that you need to have repeated and you can press zero to skip any question you prefer not to answer*
23	BP and Diabetes	Have you EVER been told by a doctor or other health worker that you have raised or high blood pressure or that you have hypertension? If YES, press 1. If NO, press 3.
24	BP and Diabetes	In the last two weeks, have you taken any drugs or medication for raised or high blood pressure that was prescribed to you by a doctor or other health worker? If YES, press 1. If NO, press 3.
25	BP and Diabetes	Have you EVER been told by a doctor or other health worker that you have raised or high blood sugar or that you have diabetes? If YES, press 1. If NO, press 3.
26	BP and Diabetes	In the last two weeks, have you taken any drugs or medications such as insulin for diabetes that was prescribed to you by a doctor or other health worker? If YES, press 1. If NO, press 3.
	Physical Activity	*Next I am going to ask you about the time you spend doing different types of physical activity in a typical week. Please answer these questions even if you do not consider yourself to be a physically active person. Think first about the time you spend doing* ***work****. Think of work as the things that you have to do such as paid or unpaid work, study/training, household chores, harvesting food or crops, fishing or hunting for food, seeking employment. In answering the following questions 'vigorous-intensity activities' are activities that require hard physical effort and cause large increases in breathing or heart rate*
27	Physical Activity	Does your work involve vigorous-intensity activity that causes large increases in breathing or heart rate like carrying or lifting heavy loads, digging or construction work for at least 10 minutes continuously? If YES, press 1. If NO, press 3.
28	Physical Activity	In a typical week, on how many days do you do vigorous-intensity activities as part of your work?
29	Physical Activity	How much time do you spend doing vigorous-intensity activities at work on a typical day? I will ask you to enter hours followed by minutes. Please enter between 16 & 0 hours now?
30	Physical Activity	Now enter between 59 & 0 minutes .
31	Physical Activity	Now think about all the moderate-intensity activities that require MODERATE physical effort and cause small increases in breathing or heart rate.<br>Does your work involve MODERATE-intensity activity that causes small increases in breathing or heart rate such as brisk walking or carrying light loads for at LEAST 10 minutes continuously? If YES, press 1. If NO, press 3.
32	Physical Activity	In a typical week, on how many days do you do moderate-intensity activities as part of your work?
33	Physical Activity	How much time do you spend doing moderate-intensity activities at work on a typical day? I will ask you to enter hours followed by minutes. Please enter between 16 & 0 hours now?

34	Physical Activity	Please enter between 59 & 0 minutes now?
	Physical Activity	*The next questions exclude the physical activities at work that you have already mentioned. Now I would like to ask you about the usual way you **travel to and from places**. For example to work, for shopping, to market, to place of worship.*
35	Physical Activity	Do you walk or use a bicycle for at least 10 minutes continuously to get to and from places? If YES, press 1. If NO, press 3.
36	Physical Activity	In a typical week, on how many days do you walk or bicycle for at least 10 minutes continuously to get to and from places?
37	Physical Activity	How much time do you spend walking or bicycling for travel on a typical day?<br>I will ask you to enter hours followed by minutes. Please enter between 16 & 0 hours now?
38	Physical Activity	Please enter between 59 & 0 minutes now?
	Physical Activity	*The next questions exclude the work and transport activities that you have already mentioned. Now I would like to ask you about sports, fitness and **recreational activities***
39	Physical Activity	Do you do any vigorous-intensity sports, fitness or recreational or leisure activities that cause large increases in breathing or heart rate like running or football for at least 10 minutes continuously? If YES, press 1. If NO, press 3.
40	Physical Activity	In a typical week, on how many days do you do vigorous-intensity sports, fitness or recreational or leisure activities?
41	Physical Activity	How much time do you spend doing vigorous-intensity sports, fitness or recreational activities on a typical day? I will ask you to enter hours followed by minutes. Please enter between 16 & 0 hours now?
42	Physical Activity	Now enter between 59 & 0 minutes.
43	Physical Activity	Do you do any moderate-intensity sports, fitness or recreational or leisure activities that cause a small increase in breathing or heart rate such as brisk walking, cycling, swimming, volleyball for at least 10 minutes continuously? If YES, press 1. If NO, press 3.
44	Physical Activity	In a typical week, on how many days do you do moderate-intensity sports, fitness or recreational or leisure activities?
45	Physical Activity	How much time do you spend doing moderate-intensity sports, fitness or recreational or leisure activities on a typical day? I will ask you to enter hours followed by minutes. Please enter between 16 & 0 hours now?
46	Physical Activity	Now enter between 59 & 0 minutes
